# Fractal density and singularity analysis of heat flow over ocean ridges

**DOI:** 10.1038/srep19167

**Published:** 2016-01-13

**Authors:** Cheng Qiuming

**Affiliations:** 2 State Key Lab of Geological Processes and Mineral Resources, China University of Geosciences, Beijing 100083, Wuhan 430074, China; 1 Earth and Space Science and Engineering, York University, Toronto, M3J1P3, Canada

## Abstract

Peak heat flow occurs at mid-ocean ridges and decreases with the age of the oceanic lithosphere. Several plate models, including the Parsons and Sclater (PSM) model, Global Depth and Heat (GDH1) model and Constant Heat flow Applied on the Bottom Lithospheric Isotherm (CHABLIS) model, have been used to predict heat flow in the ocean lithosphere. The discrepancy between the predicted and measured heat flow in the younger lithosphere (i.e. younger than 55 Myr) influenced by local hydrothermal circulation has been used to estimate hydrothermal heat flux and investigate hydrothermal processes. We can modify the cooling models by substituting the ordinary mass density of lithosphere by fractal density with singularity. This new model provides a modified solution to fit the observed heat flow data used in other models in the literature throughout the age range. This model significantly improves the results for prediction of heat flow that were obtained using the PSM, GDH1 and CHABLIS models. Furthermore, the heat flow model does not exhibit special characteristics around any particular age of lithosphere. This raises a fundamental question about the existence of a “sealing” age and accordingly the hydrothermal flux estimation based on the cooling models.

Hydrothermal systems at mid-ocean ridges involve complex cascade systems of heat transfer from the Earth’s interior to the ocean. High-temperature axial hydrothermal systems, including black smokers in oceanic spreading centres and back-arc basins have attracted much attention from researchers^1^,^2^,^3^,^4^. The heat flow decreases with distance from mid-ocean ridges^5^,^6^,^7^. Gravity and crustal thickness have been used to constrain thermal contraction models, but the variation of heat flow and see-floor depth with age or distance became the primary constraint on models of the thermal structure and evolution of the oceanic lithosphere^8^,^9^. Several possible cascade processes have been discussed to interpret the generation and evolution of heat flow, including mantle convection^10^, heat conduction^11^, circulation^12^, heat released during exothermal serpentinisation reactions^13^, heat sources in plumes and magmatic eruptions^14^,^15^, dike injection^16^ and earthquake swarms^17^. Several mathematical models have been developed for predicting spatial and temporal variations in heat flow with age and sea-floor depth. Three types of deterministic conductive models and their adaptations have been developed based on flow conduction dynamics and treatment of the lithosphere as a plate with regular boundary conditions. One model assumes that the lithosphere behaves as the cold upper boundary layer of a cooling half-space, known as Half Space cooling Model (HSM)^18^, the second model represents the lithosphere as a cooling plate with an isothermal lower boundary^6^,^19^,^20^, and the third model involves a constant flow applied on the bottom lithospheric isotherm^21^,^22^. These three types describe the general trend of the heat-flow curve. Parsons and Sclater^20^ developed a model (PSM) involving a 125-km-thick plate with a basal temperature of 1350 °C to fit the observed data. The simple cooling model predicts a linear relation between seafloor depth and t^1/2^, and heat flow and 1/t^1/2^, where t is the age of the ocean floor. PSM model assumes the same t^1/2^ dependence for sufficiently young ocean floor. For large ages these relations break down, and depth and heat flow decay exponentially to constant value^20^. However, it is known that the model can result in under predicting heat flow in old lithosphere and over-prediction of heat flow in young age lithosphere (i.e. <50 Myr)^23^,^24^,^25^,^26^. Ascendant attempts to develop new models or new boundary conditions have led to improved prediction of models to the observation. Stein and Stein^8^ developed a global depth and heat model (GDH1) using information estimated based on improved heat-flow data. The GDH1 model provides results significantly better than those from the PSM model, particularly with regard to heat flows from the older lithosphere that were previously treated as anomalies in the PSM model. The Constant Heat Flow Applied on the Bottom Lithospheric Isotherm (CHABLIS) model^21^,^22^ assumes that there is a heat flux into the base of the oceanic lithosphere from the underlying asthenosphere. This modification introduces new model with solutions associating heat flow with age as c/√t + (1 − b) and seafloor depth as −α√t + bt, where α, b and c are constants. The function for seafloor depth does not follow a square root of age law, contrary to what was usually believed then, but the function for heat flow is a power-law function similar to the square root of age except with a constant transformation. These functions fit the observed young age topography and observed old age heat flow well. These three models (PSM, GDH1 and CHABLIS) have been validated by many authors using various types of observed data and they have been often employed as standard models for comparisons^3^,^8^,^9^,^25^,^26^,^27^,^28^,^29^,^30^,^31^,^32^,^33^.

This being said, it has been a longstanding issue that the predicted heat flow values are significantly higher than observed heat flow in young age lithosphere (<50 Myr^23^, <25 Myr^29^). A lot of attention has also been directed to explaining the discrepancy between the predicted and observed heat flow^33^,^34^,^35^,^36^,^37^,^38^,^39^. One common justification for the discrepancy is that the measurements of heat flow near ridge crests are not truly representative due to the lack of thick sedimentary cover within ~100 km of the ridge crest^23^. The sediment thickness was used as either a filter to select subset of heat flow dataset or to correct heat flow data^25^,^28^,^35^,^36^,^37^. The over-prediction is thought to result from the transport of significant amounts of heat by water circulation^38^ rather than by the conductive cooling assumed in such models. These heat-balance models indicate that the heat input to oceanic ridges resulting from lithospheric creation and seafloor spreading is less than the rate of hydrothermal heat loss, even at rapidly spreading ridges^25^,^39^. Due to the heat flow anomalies observed in the young seafloor, the data from the younger lithosphere is usually excluded in cooling models calibration. In addition, two different types of functions are usually utilized to fit the heat flow data within two separate age ranges: a square root function of age for young seafloor and an exponential function for old seafloor^20^,^21^,^25^,^29^. Under the assumption of hydrothermal circulation, the discrepancy between predicted and observed heat flow in young seafloor can be used to estimate the level of hydrothermal heat flux and power loss^3^,^9^,^25^,^27^,^30^,^31^,^32^. The drawback of this bi-function model is that the discontinuity of the change rate (derivatives) of heat flow versus age might be difficult for interpretation and the different choice of the cut-off age separating two age domains may cause bias in estimation of heat flux.

While the heat flow solutions of all three models, PSM, GDH1 and CHABLIS have singularity at the zero age, the common explanation about the cause of singularity is due to discrepancy between boundary conditions at the ridge crest where both depth and age are zero^19^,^20^,^40^. A model assuming a non-uniform temperature distribution at the ridge crest as initial condition was proposed which yields predicted heat flow curves free of a singularity^40^. In that model it introduced a new parameter 1/m representing the proportion of the upper cooling part of lithosphere in which the temperature reduced from T_1_ to T_0_ at the surface. 1/m can also be reviewed as relative depth of heat source in respect to the total thickness of lithosphere. This thermal model allows one to estimate the depth of the cooling below ridge crest (excluding zero) from heat flow measurement. If 1/m approaches to zero or m to infinity, the predicted heat flow solution reduced to the same as obtained by PSM model. Since the mid-ridge is usually considered the place where the asthenosphere and lithosphere meet, the cooling depth at the ridge can be thought as close to zero, thus the solution with singularity at the zero age. In the current paper, we will show that the fractal density of seafloor around the ridge crest can also cause singularity which has never been accounted previously in heat flow modelling. The singularity affects the cooling model especially the solution valid in the young age seafloor. It will show that account of the effect of fractal density will reduce the divergence between observed heat flow and the predicted heat flow by cooling models.

According to the new model in which the ordinary density of lithosphere is substituted by fractal density, the heat flow function becomes a single power-law model, ∝t^−1/2−Δα^, which can be interpreted as a product of two components: t^−1/2^ and t^−Δα^, the former corresponding to the solution of standard HSM with constant model parameters, and the latter being related to the singularity of fractal density of the lithosphere at the ridge. For CHABLIS model the modification can be expressed as ∝[c/√t + (1 − b)]t^−Δα^ ∝ t^−1/2−Δα^ when t →0 if Δα < 0 (as it will be shown in discussion section). This modification on heat flow models will be discussed and evaluated with various datasets used in the literature.

## Results

Several global datasets have been used in the literature for plate cooling model validation and construction. For example, the PSM model was constrained using about 3300 heat flow determinations^20^, GDH1 by about 5500 data^8^ and more recently these models were re-evaluated by Hasterok *et al.*^28^ using an updated global heat flow database including more than 14,000 heat flow determination. In the above works, the cooling models are usually fitted with two functions with age separation at various cut-off values, such as 120 Myr in PSM^20^, 55 Myr in GDH1 model^25^, 80 Myr in CHABLIS model^21^ and 48.1 Myr in Hasterok’s model (named H model herein)^29^. Although these models ensure two functions fitted to the observed data in two age ranges exhibit continuity at the cut-off age, the two separate models are irrational in the sense that the derivatives of the functions with respect to age are discontinuous. Several recent studies used the updated database^27^ and subsets by filtering or sediment-correction in model calibration^3^,^9^,^27^,^28^,^29^,^30^,^31^,^32^. A few sets of site specific data in young seafloor, such as heat flow measured in bare rocks have also been applied^29^,^41^. Hasterok^29^ used filtered and corrected values from a large database to evaluate several models and suggested a new H model which significantly reduces the issue of hydrothermal deficit on <25 Myr seafloor. Unfiltered data for old age seafloor, filtered data and several high resolution site-specific heat flow data in young age seafloor were further combined to form a high quality dataset with minimal hydrothermal disturbance for model calibrations^30^.

Here, I utilize the above datasets used in the literature for PSM, GDH1 and H models to evaluate the new power-law model for heat flow derived on the basis of fractal density. The details of the derivation of the model will be described in the *Methods* section. It will be demonstrated that the power-law model, ct^−(1/2+Δα)^, where c is a constant and Δα is the singularity of fractal density of lithosphere, fits the observed heat flow datasets very well throughout the observed age range. The implication of fractal density inclusion into heat flow modelling will be presented in the *Discussion* section.

### The data used by Stein and Stein^8^

**Figure 1:**
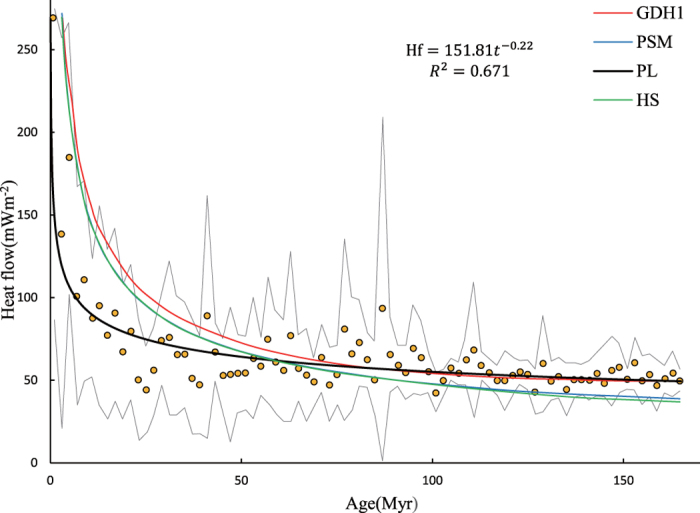
Observes heat-flow data fitted using mathematical models. Heat flows are from sites in the North Pacific (north of Equator) and Northwest Atlantic. The averaged data in 2-Myr bins are denoted by dots, and the standard deviation about the mean is denoted by the envelope. Red curves denote the results fitted using the Parsons and Sclater model (PSM), a cooling half-space model (HS), and the GDHl plate model (after Stein and Stein^8^). The solid black curve denotes the fit derived from the model presented here.

Stein and Stein^8^ compiled 5,539 data points at sites younger than 166 Myr, with good coverage of the main oceanic plates. Figure 1 shows the averaged heat flow data in 2-Myr bins (as dots) and one standard deviation concerning the mean value. The data was fitted using the PSM and GDH1 plate models. To reanalyse the heat flow data using the power-law model introduced in the current paper, the 83 dots were digitised to be converted to numerical data and were then fitted using the least-squares method. The results include the function q(t) = 151.81t^−0.22^, a coefficient of determination R^2^ = 0.671, and a Student’s t-value = 8.1 (n = 83 samples). The results show that the power-law model with an exponent −0.22, or Δα = −0.28 fits the observed heat flow data very well throughout the age range. Unlike the results obtained by other models such as PSM, GDH1, CHABLIS and H models, the single power-law model fitted to the data over the whole age range does not exhibit special characteristics around any particular age, such as t = 55 Myr. Any order-derivatives of the power-law heat flow function are continuous everywhere except at t = 0, where heat flow exhibits a singularity.

### The data used by Stein and Stein^25^

**Figure 2:**
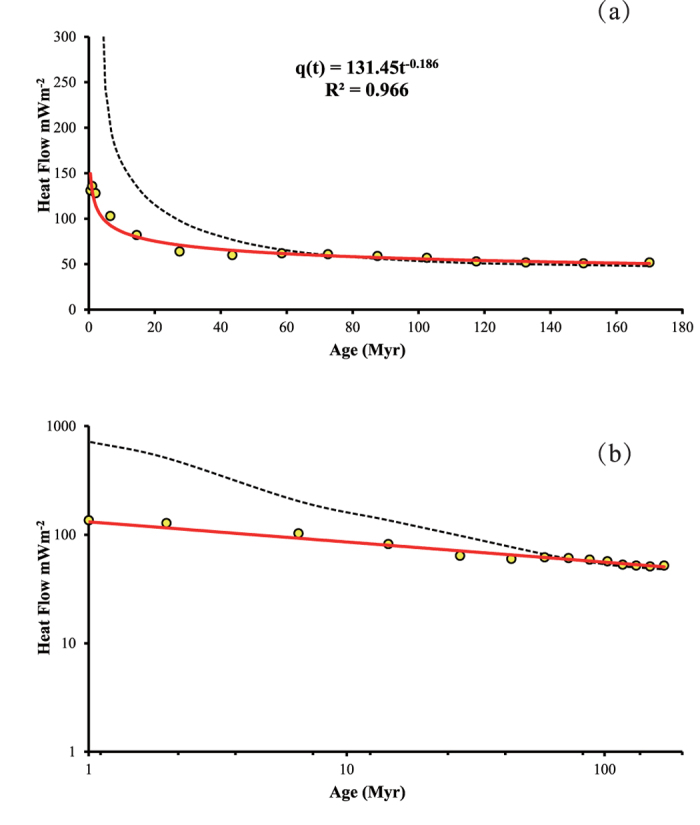
Data shows (a) heat flow vs. age and (b) log-log scale plot. The dots represent the measured data (from Stein and Stein^25^), the dashed line denotes the fitted curve obtained using the GDH1 model and the solid lines symbolize the fitted curves obtained using the presented model.

The second dataset is the averaged heat flow data used in Stein and Stein^25^. The data is the same dataset as used by Stein and Stein^8^ except the average heat flow calculated in 15 variable unequal age bins including several small bins younger than 6 Myr. Several of the individual values were obtained by averaging the results of detailed surveys on crust younger than 6 Myr to avoid biasing the data. The data is shown in Fig. 2a,b.

The data were fitted using the least-squares method as well as the power-law model. The equation is shown in Fig. 2a. The results (Fig. 2a,b) show that the power-law function with exponent −0.186, or Δα = −0.314, fits the heat-flow data very well throughout the age range, with R^2^ = 0.966 and Student’s t-value = 13.5.

### The data used by Hasterok *et al.* 28

**Figure 3:**
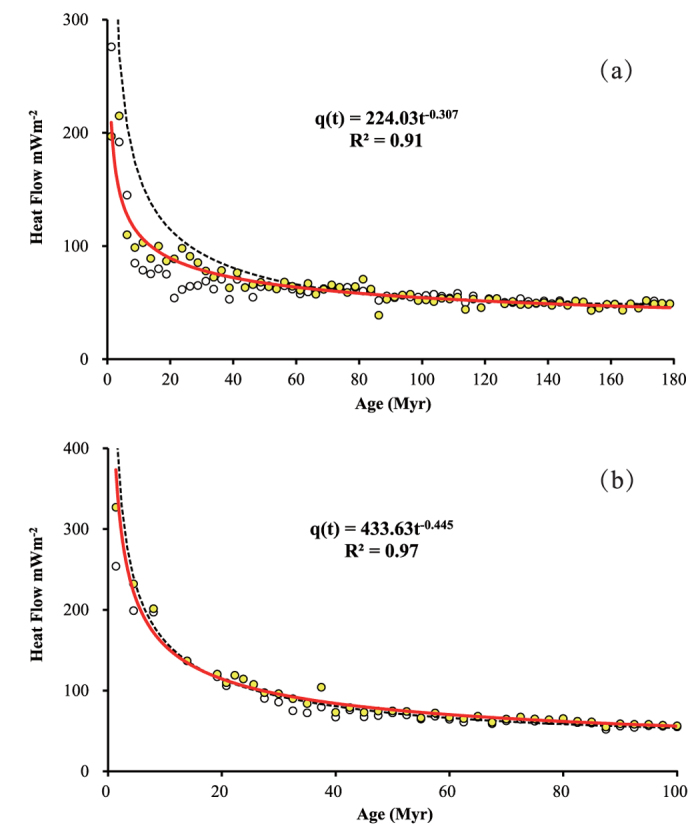
Data shows heat flow vs. age. (**a**) The empty dots and yellow dots represent the unfiltered and filtered mean heat flow data per 2.5 Myr bin, respectively (Table 1 of Hasterok *et al.*^28^). (**b**) The white and yellow dots represent the uncorrected and corrected median heat flow per 2 Myr bin. The data points include unfiltered, filtered and site-specific heat flow data (Table 1 of Hasterok^29^). The dashed lines denote the fitted curve obtained using the GDH1 model. The solid lines denote the fitted curves obtained using the model presented here. The age range for model fitting to the median data is <100 Myr.

The third dataset is from Hasterok^28^ who have compiled the most updated and complete heat flow database nearly 15,000 oceanic heat flow measurements. Based on the database, the authors created a new heat flow model which was further used to estimate the conductive heat flow loss through the seafloor. To minimize the effect of ventilated hydrothermal circulation on heat flow and maximize the number of data points used in model fitting, a subset of heat flow measurements was extracted by filtering with >400 m of sediment cover and located >60 km from the nearest seamount. In total, 5023 measurements were retained which is about 35% of the initial pre-filtered dataset. The effect of hydrothermal circulation on average heat flow obtained with the new dataset is substantially reduced (Fig. 3a)^29^. However, a small heat flow deficit compared to lithosphere cooling models persists as ages <50 Myr, especially <25 Myr. It was interpreted that some of this deficit could result from incomplete thermal rebound and uncertainties in sediment thickness^29^. A combined dataset including unfiltered, filtered, and site-specific data was used to improve goodness of fit to GDH1 model (Fig. 3b) not only for heat flow but also for seafloor depth^29^. In this section, I will use the heat flow datasets^28^,^29^ to evaluate the power-law model.

Both the average heat flow per 2.5 Mys bin calculated from the filtered and unfiltered data^28^ are shown in Fig. 3a. The data were fitted using the least-squares method in accordance with the power-law model. The results show that the power-law function fits the average heat flow data very well throughout the age range. The curves fitted to unfiltered data (not shown) and filtered data result in, q(t) = 186.57 t^−0.271^ with R^2^ = 0.81 for unfiltered data, and q(t) = 224.03 t^−0.307^ with R^2^ = 0.91 for filtered data, respectively. Accordingly, the fractal density singularity are calculated as Δα = −0.229 and Δα = −0.193, respectively.

Figure 3b shows the results based on the median heat flow per 2 Myr bin calculated from a combined dataset with filtered, unfiltered and site-specific measurements. Considering the skewed heat flow data distribution and test of importance of choose of non-Gaussian versus Gaussian statistics on the dataset, the median rather than mean statistics were used on assessment of heat flow distribution and estimation of the total power deficit^30^. With the median heat flow data, the least square fit with power-law function is well up to 100 Myr. There would be a slight under-estimation if the power law function is extrapolated to fit the data for whole age range 180 Myr. The median heat flow data from combined unfiltered, filtered and site-specific measurements <100 Myr were fitted and shown in Fig. 3b, which gives q(t) = 433.63 t^−0.445^ and Δα = −0.055 with R^2^ = −0.97.

## Discussions

The three forgoing examples indicate that peak heat flow measured over the oceanic ridges exhibit singular behaviour, following a power law relationship with age or distance from the ridges. The decay trend of heat flow with age can be approximately described by power-law models with negative exponent (−1/2 − Δα). The exponent was estimated ranging from −0.186 to −0.307 for average heat flow within 2 or 2.5 Myr bins and −0.445 calculated for median heat flow per 2 Myr bin. The single power-law model fits the heat flow data in each example throughout the available age range (except for median heat flow data with correction in age range younger than 100 Myr). The results suggest that there are no specific characteristics of measured heat flow at any particular age. The power-law function is continuous, and any order-derivatives of power-law function are also continuous except at age zero at which it exhibits singularity. According to separation of active hydrothermal circulation near the ridge axis and passive circulation away from ridges^25^,^42^,^43^ the current result may question the presence of a “sealing” age or simply gradual change from a predominantly “active” to “passive” phenomena as the lithosphere age increases. Stein and Stein^25^ concluded hydrothermal flow decreases with age due to reduced crustal porosity and permeability. The heat flow fraction depends primarily on crustal age and secondarily on sediment distribution; it increases with age because of reduced flow in the crust due to decreased crustal porosity and hence permeability^25^,^44^. The overlaying sediment may reduce, but not eliminate the effects of water flow. Therefore, this result indicates that, if it exists, the “sealing” age, representing the age at which the hydrothermal heat flux becomes minor, may not cause abrupt change in heat flow pattern with, for example, discontinuity of derivatives of the heat flow function.

The three examples used for analysis all give negative singularity of fractal density (Δα ∼ −0.314 to −0.193 for average heat flow and −0.055 for median heat flow) implying significant negative anomalies of fractal density around ridges. The variation of the singularity Δα around value −0.25 estimated from these datasets is due to selection and filtering/correction of the heat flow data. It is also affected by the decision of averaging of data with different scheme of partition the age intervals. For example, in the first example, equal interval of 2.5 Myr was used to calculate average heat flow which gives relatively more data points in old age seafloor than young age seafloor. Therefore the least squares fit would be influenced by the points in old age seafloor. This gives a relatively small singularity Δα = −0.28. In contrast, the second dataset uses small number of variable unequal age bins (15 bins) including several small bins younger than 6 Myr, this dataset gives relatively more influence of young data on the least square fitting which can lead to larger value of singularity Δα = −0.314. The number of data and data quality in the dataset can also affect the estimation of the model, for example, the third dataset with far more data points gives singularity value Δα = −0.229 for unfiltered data and Δα = −0.193 for filtered data. The reduced value of singularity estimated from the filtered dataset is understandable since it has few data points from relatively young age which in turn increase the influence of data from relative old age. In general, more restrictive filters applied to heat flow measurements lessen the effect of heat flow data with young age on the heat flow modelling, consequently the weaker the singularity value. This is common in fractal and multifractal modelling for estimating multiple scale singularities that the actual values of singularity could be affected by the range of scale used for calculation. Future study may need to focus on individual ridges to explore the spatial variation of fractal density calculated from heat flow data from different oceans. Ideally, the singularity of fractal density of the crust should be independently estimated by associating average density and scale from measurements about density data of lithosphere. The singularity of fractal density can then be used in the cooling model as parameter. However, to estimate singularity of fractal density of lithosphere over ridges one needs a large number of samples taken from the surface and subsurface to provide database to estimate fractal density considering 3D heterogeneity of lithosphere around ridges. An indirect way to characterize the lithosphere’s density might be by inversion of gravity or seismic data since these measurements are related to the density of the lithosphere. These types of data are also affected by other factors. The spatial distributions of oceanic topography and lithosphere density have been found to exhibit multifractality with multiple scale singularities^45^. However, these types of multifractality of lithosphere density have not been considered in heat flow dynamic modelling.

Several authors have discussed the influence of gravity and density of the lithosphere on hydrothermal circulation occurrences in lithosphere over ridges. For example, previous near-bottom gravity data indicated a porosity of 30% over the first few hundred meters of the upper layer of the oceanic crust^41^. High porosity is supported by low seismic velocities^32^. High porosity upper crustal around ridges is also reflected by the low mass density of lithosphere which can be indirectly reflected by negative free-air gravity anomalies. Studies^46^,^47^,^48^ have demonstrated that the gravity around the oceanic ridges and transform faults are incredibly complex with positive and negative residual mantle Bouguer gravity anomalies. The mass density of lithospheric over these areas has significant variability due to change of rock porosity, serpentinization of mantle peridotite, faults and fractures, and/or crustal thickening. Watts^46^ stated that the most striking of the features found in gravity anomalies over oceanic rifts are large amplitude free-air gravity anomaly low over oceanic rifts in pacific, Indian, and Atlantic oceans. Lambeck *et al.*^49^ reported that gravity anomalies over ridges can be explained by the thermal expansion of the lithosphere at the ridges, suggesting that gravity measurements can be used as constraints on the lithospheric models in the same way as heat flow and topography measurements. To summarize, the above suggestions indicate that the lithosphere around ocean ridges, especially the uppermost crust, is very complex not only in porosity, mass density, composite, topography, seismic and gravity properties, and texture and structures but also the irregular geometry and multi-scale of hydrothermal circulations within the lithosphere. Some but not all of these properties have been accounted in heat flow modelling. It was commented that the deviations of measured heat flow from the plate model prediction may be due to temperature and pressure effects on physical properties/processes not accounted for by the plate model such as lithospheric strength, heat production, and thermal rejuvenation^28^. Understanding the hydrothermal flow process should ideally reconcile models based on large-scale average crustal properties, such as heat flow or seismic velocity, with site measurement of water flow and crustal physical property^25^. The concept of fractal density proposed in this paper can describe the non-linear scaling property of mass density and characterize the complexity of density with change of support. The new solution derived from the standard conductive cooling model with assumed fractal density may consist of two components: heat flow solution with fractal density ρ_α_ and other constant parameters (such as ∝t^−1/2^) and the component with singularity of fractal density (∝t^−Δα^). As explained in the *Methods* section, the singularity index Δα has three possibilities: if Δα = 0, corresponding to ordinary non-singular density, then the solution of cooling model remains unaffected; if Δα > 0, corresponding to “convex” anomaly of density over ridge, then the solution of cooling model will show an enlarged exponent (0.5 + Δα > 0.5), in this case the measured heat flow tend to deviate from the ordinary solution; and if Δα < 0, corresponding to “concave” anomaly of density over ridge, then the new solution of cooling model will show a reduced exponent (0.5 + Δα < 0.5), in this case the measured heat flow tend to converge to the ordinary solution.

It must be mentioned that the value of singularity of fractal density should be estimated separately from the heat flow measurement so that it can be used to assess the effect of fractal density on the prediction of heat flow by cooling models, and accordingly to judge the influence of hydrothermal circulation on heat flow prediction. Without a clear understanding and good estimate of the singularity of fractal density, it is impossible to differentiate the influence of hydrothermal flow flux and the influence of fractal density. Another complication of this issue is that the singularity of fractal density may vary from ridge to ridge; therefore, fractal density should be investigated in various specific regions around ridges. Considering this, the hydrothermal flux’s estimation conducted in the literature based on ordinary cooling model and measurements of heat flow can be questioned. Nevertheless, introduction of fractal density into cooling model for heat flow prediction in ocean ridge might have opened a path for future research. I need to point out that the work about insertion of fractal density in cooling model of the current paper is only introduced for modification of heat flow modelling. Future study should also take into account influence of fractal density on substance of floor which is an important constraint of cooling model.

## Methods

Non-linear processes are widespread in nature. Extreme physical, chemical and biological processes occurring in the Earth’s crust often result in a singularity of energy release or mass accumulation^50^. In this paper, we show that the end products of these types of singular processes such as hydrothermal systems at mid-ocean ridges can be characterised as fractal densities based on a power-law relationship between density and scale.

### Fractal density and power-law model

Since density is a fundamental physical parameter involved in the thermal models (PSM, GDH1 and CHABLIS) used for prediction of heat flow at the mid ocean ridges, we will first introduce a new definition of fractal density with nonlinear property which was ignored by the traditional dynamics systems. The principle of density was discovered by the Greek scientist Archimedes approximately 2000 years ago. Density has become a foundational property of mass and energy and a well-known physical concept with a variety of applications in nearly all fields of study. The density of material or energy is defined as its mass or energy per unit volume. Therefore, density often has units of mass over volume (e.g., g/cm^3^, kg/m^3^) or energy over volume (J/cm^3^, w/L^3^).

To calculate the mass density of an object (ρ), one takes the mass contained in a volume (m(v)) divided by the volume (v):


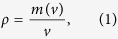


where ρ is the average density of an object, which becomes independent of the volume only if the density of the object is uniform. However, if the object has heterogeneous properties, the density must be calculated using the derivative of the mass over volume:


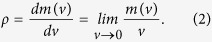


The above density exists only if the limit converges when the volume becomes infinitesimal. In this paper, we will demonstrate that the limit in Eq. (2) does not always converge for complex objects with fractal properties. A new concept of fractal density is defined here as the limit of the following relation (3) if there is a parameter α (a positive value) so that the following limit converges^51^,^52^:


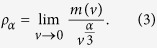


The value of ρ_α_ can be considered to be the generalised ordinary density ρ because the relation in Eq. (2) becomes a special case of Eq. (3) when α = 3. The fractal density defined in Eq. (3) has units of the mass ratio to a fractal set of α dimensions, for example, g/cm^α^ or kg/m^α^. Similarly, the units of fractal energy density can be J/cm^α^ or w/L^α^. Combining Eqs. (2) and (3) yields the following relationship between the ordinary density and the fractal density:


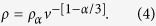


Thus, the ordinary density obeys a power-law relationship with the volume with the following properties^51^,^52^:


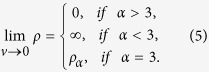


In accordance with these properties, the ordinary density shows a singularity when α ≠ 3. The concept of fractal density and Eqs. (3) and (4) can be extended to other situations, for example, areal and linear densities. A general model associating the fractal density and the ratio of mass and scale (linear size of an E-dimensional set) can be expressed as follows:


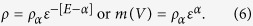


This equation indicates that both ordinary density and mass follow power-law relations of scale *ε*. A power-law function is determined by two parameters: the fractal density ρ_α_ and the exponent–singularity index α (fractal dimension), or E-α; the latter corresponds to the co-dimension of fractal density. The singularity index measures the deviation of the fractal dimension from the dimension of normal density. These two parameters (ρ_α_ and α) can be estimated from observed data by measuring the intercept and slope of the log-log plot of *m* against *ε*, which plots as a straight line. Singularity is observed in non-linear processes including cloud formation^53^, rainfall^54^, hurricanes^55^, flooding^56^, landslides^57^, earthquakes^58^, mineralisation^59^,^60^, and solar wind turbulence^61^.

### Multiplicative cascade processes and formation of fractal density

Multifractal cascade models play a fundamental role in quantifying turbulent intermittency and other non-linear processes^62^. Here, we will use a relatively simple 1-dimensional de Wijs multiplicative cascade model^63^ to demonstrate the formation of fractal density. The simple version of the de Wijs cascade model involves the partitioning of each unit segment (of length L) into two sub-segments of equal size. The measured value (m) of a quantity in the unit segment can then be written as d . m for one half and (1-*d*)·m for the other half (0 < *d* < 1) so that total mass is preserved. The coefficient of dispersion (*d*) is independent of segment size. If d > ½, the maximum measured value after *n* subdivisions is μ = *d*^*n*^m, and the minimum value is μ = (1-*d*)^*n*^m. If d < ½, the maximum and minimum values are switched. The general measured value after n subdivisions can be represented as μ = d^k^(1-d)^n-k^m, where 0 ≤ k ≤ n. The number of segments with this value is 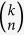. It has been proven that the measure μ(ξ) converges as n approaches infinity, and


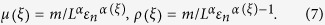


where ξ = k/n such that 0 ≤ ξ ≤ 1 and


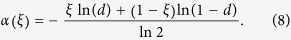


The quantity α(ξ) represents the singularity index in the context of multifractals^64^,^65^. Note that when *ε*_n_ →0, the ordinary density of bar segments with length *ε*_n_ and measures μ(*ε*_n_) approaches either infinity or zero unless α  = 1, which corresponds to d = 1-d = 0.5. In other words, if d ≠ 0.5, then α ≠ 1, and the ordinary density approaches zero if α < 1 or approaches infinity if α > 1. The dimension of fractal density varies from one location to the next along the bar L. The dimensions of the fractal densities of the two segments at the ends of the bar are α(0) = −log(d)/log(2) ≠ 1 and α(1) = −log(1-d)/log(2) ≠ 1.

### Fractal heat density and power-law model

Here we will show that “singular” locations with fractal densities where *α* < E may indicate an anomalous release of energy caused by hydrothermal events at mid-ocean ridges. According to heat-flow dynamics, the volumetric heat content (H in units of Jm^−3^) can be represented by the equation^2^


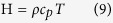


where *c*_*p*_ is the calorific capacity, ρ is the mass density, and T is the temperature. In lithosphere cooling models introduced in the literature for modelling hydrothermal event including heat flow over mid oceanic ridges, the mass density ρ is often assumed to be ordinary density either with constant value of mantle density (3.0 g/cm^3^ or 3.3 g/cm^3^) or treated as linear function of depth or nonlinear function of temperature^66^. Similarly, the values of *c*_*p*_ can be taken as constant such as 0.25 cal/g°C or nonlinear function of temperature. The standard analytical lithosphere cooling model ignores the variation of ρ and *c*_*p*_ with temperature and treats these parameters as constants. It was pointed out by McKenzie *et al.* that the effects of such variations of ρ and *c*_*p*_ with temperature are small and they are easily included in a numerical scheme^66^. The plate model with temperature dependent parameters fits the variation of heat flow and depth with age at least as good as those of the analytic model with constant and adjustable coefficients. One should be reminded that these models involve ordinary mass density ρ whether as constant value, linear function of depth or nonlinear function of temperature. For example, it can have the normal density unit of g/cm^3^. However, as pointed out in forgoing discussions of this paper, many studies indicate the density variation of lithosphere over ridges is complicated; thus, fractality of density should be considered. Several studies indicated^43^,^44^,^45^ that free-air gravity anomaly low over oceanic rifts could reflect that the mass density of lithospheric over these areas are of significant variability due to change of rock porosity, serpentinization of mantle peridotite, and/or crustal thickening. The increase of serpentinization of the uppermost mantle lithosphere can also change the density of ocean crust. The spatial distributions of oceanic topography and lithosphere density have been found of multifractality with multiple scale singularities^44^. The property of multifractals and multiple scale singularities of lithosphere density with respect to measuring scale have not yet been taken into account in any lithosphere cooling models. In this paper, we attempt to consider fractal density of lithosphere (6), the volumetric heat content in a volume of linear size *ε* should obey the relationship


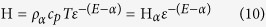


where H_α_ is a fractal thermal density with units of J/m^α^, and, accordingly, the ordinary thermal density H should contain a component expressed as power-law relationship of scale *ε*. Given the new definition of fractal density and fractal heat density, the heat flow q expressed in units of volume can be rewritten


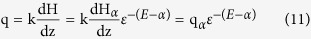


where k is the thermal diffusivity which has been treated as constant in the standard analytic model and as a function of temperature in numerical modeling^66^, and q^α^ is the heat flow calculated based on the fractal heat density. Therefore, the ordinary heat flow can be treated as the average heat flow per unit volume, obeying the power-law relationship with the volume. This relation (11) indicates that the ordinary heat flow q should be modified by the term tackling the singularity of the fractal density.

According to the prevailing paradigm of the PSM, the GDH1 and CHABLIS that model the heat flow measured at the surface of a mid-ocean ridge can be described by a one-dimensional function q(t) where t is the age of the lithosphere. The distance from the ridge x can be calculated from the age of the lithosphere according the constant speeding rate. Accordingly, q(t) can be expressed either as the distance from the ridge or the lithosphere’s age. Considering the one-dimensional problem, the scale *ε* can be treated as two times that of x, *ε* = 2x. Combining the ordinary heat flow function, q_α_(t), to be derived from the standard plate model with constant k and *c*_*p*_ except that fractal density ρ_α_ is in the form of singularity (11), we can derive the new function as


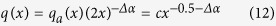


Consequently, the power-law model (12) can be formulated as a function of age with exponent −(0.5 + Δα) (Δα = E − α, E = 1 for one dimensional problem). For CHABLIS model the function (12) becomes approximation when x → 0 and Δα < 0. This function was fitted to the datasets in Figs 1 - 3 and they give estimated value Δα = −0.28, −0.314, −0.229, −0.193 and −0.055, respectively. These values are consistently less than zero, indicating strong negative singularity of mass density of lithosphere would be expected over the ocean ridge.

## Additional Information

**How to cite this article**: Qiuming, C. Fractal density and singularity analysis of heat flow over ocean ridges. *Sci. Rep.* **6**, 19167; doi: 10.1038/srep19167 (2016).

## References

[b1] Baker E. T. & Garman C. R. On the global distribution of hydrothermal vents, in The Thermal Structure of Oceanic Crust and the Dynamics of Hydrothermal Circulation. Ed. By Garman C. R., Lin J. & Parsons L. Hydrothermal Interaction between the Lithosphere and Oceans. Geophys. Monog. Series 148, 245–266 (2004).

[b2] Lowell R. P. & Germanovich L. N. Hydrothermal processes at mid-ocean ridges: results from scale analysis and single-pass models. Ed. By Garman C. R., Lin J. & Parsons L. Hydrothermal Interaction between the Lithosphere and Oceans. Geophys. Monog. Series 148, 219–244 (2004).

[b3] Samuel H. & King S. D. Mixing at mid-ocean ridges controlled by small-scale convection and plate motion. Nat. Geosci. 10.1038/NGEO2208 (2014).

[b4] Hamblin W. K. & Christiansen E. H. Earth’s Dynamic Systems, 10th Edition, Prentice Hall. 816pp (2003).

[b5] Von Herzen R. P. & Uyeda S. Heat flow through the eastern Pacific ocean floor. J. Geophys. Res. 68, 4219–4250 (1963).

[b6] Langseth M. G., Le Pichon X. & Ewing M. Crustal structure of the mid-ocean ridges: 5. Heat flow through the Atlantic Ocean floor and convection currents. J. Geophys. Res. 71, 5321–5355 (1966).

[b7] Sclater J. G. & Francheteau J. The implications of terrestrial heat flow observations on current tectonic and geochemical models of the crust and upper mantle of the Earth. Geophys. J. Roy. Astron. Soc. 20, 509–542 (1970).

[b8] Stein C. A. & Stein S. A model for the global variation in oceanic depth and heat flow with lithospheric age. Nature. 359, 123–129 (1992).

[b9] Goutorbe B. & Hillier J. K. An integration to optimally constrain the thermal structure of oceanic lithosphere. J. Geophys. Res. 118, 432–446 (2013).

[b10] Parsons B. & McKenzie D. Mantel convection and the thermal structure of the plates. J. Geophys. Res. 83, 4485–4496 (1978).

[b11] England P. C. & Richardson S. W. Erosion and the age dependence of continental heat flow. Geophys. J. Roy Astron. Soc. 62, 421–437 (1980).

[b12] Mottl M. J. & Wheat C. G. Hydrothermal circulation through mid-ocean ridge flanks: Fluxes of heat and magnesium, Geochim. Cosmochim. Acta 58, 2225–2237 (1994).

[b13] Kelley D. S., Baross J. A. & Delaney J. R. Volcanoes, fluids, and life at mid-ocean ridge spreading centres. Annul. Rev. Earth Planet. Sci. 30, 385–491 (2002).

[b14] Richardson S. W. & Oxburgh E. R. The heat flow field in mainland UK. Nature 282, 565–567 (1979).

[b15] Baker E. T., Massoth G. J. & Feely R. A. Cataclysmic hydrothermal venting on the Juan de Fuca Ridge. Nature 329, 149–151 (1987).

[b16] Lowell R. P. & Germanovich L. N. Dike injection and the formation of megaplumes at ocean ridges. Science 267, 1804–1807 (1995). 17775809 10.1126/science.267.5205.1804

[b17] Johnson H. P. *et al.* Earthquake-induced changes in a hydrothermal system at the Endeavour Segment, Juan de Fuca Ridge, Nature 407, 174–177 (2000). 11001052 10.1038/35025040

[b18] Davis E. E. & Lister C. R. B. Fundamentals of ridge crest topography. Earth. Planet. Sci. Lett. 21, 405–413 (1974).

[b19] McKenzie D. P. Some remarks on heat flow and gravity anomalies. J. Geophys. Res. 72, 6261–6273 (1967).

[b20] Parsons B. & Sclater J. G. An analysis of the variation of ocean floor bathymetry and heat flow with age. J. Geophy. Res. 82, 803–827 (1977).

[b21] Doin M. P. & Fleitout L. Thermal evolution of the oceanic lithosphere: an alternative view. Earth. Planet. Sci. Lett. 142, 121–136 (1996).

[b22] Crough S. T. Thermal model of oceanic lithosphere. Nature 256, 388–390 (1975).

[b23] Oxburgh E. R. & Turcotte D. L. Increased estimate for heat flow at oceanic ridges. Nature 223, 1354–1355 (1969).

[b24] Stüwe K. Geodynamics of the Lithosphere: an Introduction. Springer. 449pp (2002).

[b25] Stein C. & Stein S. Constraints on hydrothermal heat flux through the oceanic lithosphere from global heat flow. J. Geophys. Res.: Solid Earth (1978–2012) 99, 3081–3095 (1994).

[b26] Elderfield H., Becker K., Davis E. E. Davis E. E. & Elderfield H. Foundations of research into heat, fluid, and chemical fluxes in oceanic crust, Hydrogeology of the Oceanic Lithosphere. Cambridge University Press, Cambridge, 28–56 (2004).

[b27] Grose C. J. Properties of oceanic lithosphere: Revised plate cooling model predictions. Earth. Planet. Sci. Lett. 333–334, 250–264 (2012).

[b28] Hasterok D., Chapman D. & Davis E. Oceanic heat flow: Implications for global heat loss. Earth. Planet. Sci. Lett. 311, 386–395 (2011).

[b29] Hasterok D. A heat flow based cooling model for tectonic plates. Earth. Planet. Sci. Lett. 361, 34–43 (2013a).

[b30] Hasterok D. Global patterns and vigor of ventilated hydrothermal circulation through young seafloor. Earth. Planet. Sci. Lett. 380, 12–20 (2013b).

[b31] Grose C. J. & Afonso J. C. The hydrothermal power of oceanic lithosphere. Solid Earth Discuss. 7, 1163–1207 (2015).

[b32] Weekly R. T., Wilcock W. S. D. & Toomey D. R. Upper crustal seismic structure of the Endeavour segment, Juan de Fuca Ridge from travel time tomography: Implications for oceanic crustal accretion. Geochem. Geophys. Geosy. 15, 1296–1315 (2014).

[b33] Hutnak M. *et al.* Hydrothermal recharge and discharge guided by basement outcrops on 0.7-3.6 Ma seafloor east of the Juan de Fuca Ridge: Observations and numerical models. Geochem. Geophys. Geosy. 7, Q07O02 (2006).

[b34] Jarvis G. T. & Peltier W. R. Oceanic bathymetry profiles flattened by radiogenic heating in a convecting mantle. Nature 285, 649–651 (1980).

[b35] Davis E. E., Becker K. & He J. Costa Rica Rift revisited: Constraints on shallow and deep hydrothermal circulation in young oceanic crust. Earth. Planet. Sci. Lett. 222, 863–879 (2004).

[b36] Spinelli G. A. *et al.* Hydrothermal seepage patterns above a buried basement ridge, eastern flank of the Juan de Fuca Ridge. J. Geophys. Res. 109, B01102 (2004).

[b37] Spinelli G. A. & Harris R. N. Effects of the legacy of axial cooling on partitioning of hydrothermal heat extraction from oceanic lithosphere. J. Geophys. Res. 116, B09102 (2011).

[b38] Anderson R. N. & Hobart M. A. The relation between heat flow, sediment thickness, and age in the Eastern Pacific. J. Geophys. Res. 81, 2968–2989 (1976).

[b39] Macdonald K. C., Becker K., Spiess F. N. & Ballard R. D. Hydrothermal heat flux of the “black smoker”vents on the East Pacific rise. Earth. Planet. Sci. Lett. 48, 1–7 (1980).

[b40] Lubimova E. A. & Nikitina V. N. On heat flow singularity over mid-ocean ridges. J. Geophys. Res. 80, 232–243 (1975).

[b41] Salmi M. S., Johnson H. P., Tivey M. A. & Hutnak M. Quantitative estimate of heat flow from a mid-ocean ridge axial valley, Raven field, Juan de Fuca Ridge: observations and inferences. J. Geophys. Res. 119, 6841–6854 (2014).

[b42] Lister C. R. B. “Active” and “passive” hydrothermal systems in the oceanic crust: predicted physical conditions. In The Dynamic Environment of the Ocean Floor (Eds. Fanning K. A. & Manheim F. T.). Lexington Books, Gomer Publishing, 441–470 (1982).

[b43] Fehn U. & Cathles L. M. The influence of plate movement on the evaluation of hydrothermal convection cells in the oceanic crust. Tectonophysics 125, 289–312 (1986).

[b44] Anderson R. N., Langseth M. G. & Sclater J. G. The mechanisms of heat transfer through the floor on the Indian Ocean. J. Geophys. Res. 82, 3391–3409 (1977).

[b45] Lovejoy S., Schertzer D. & Gagnon J.-S. Multifractal simulations of the earth’s surface and interior: anisotropic singularities and morphology. *In*: Cheng Q. & Bonham-Carter G. (eds) GIS and Spatial Analysis: *Proceedings of IAMG’05: The Annual Conference of the International Association for Mathematical Geology*, Toronto, August 21-25,2005, International Association for Mathematical Geology, Kingston, Ontario 1, 37–54 (2005 August).

[b46] Gregg P. M., Lin J., Behn M. D. & Montési L. G. J. Spreading rate dependence of gravity anomalies along oceanic transform faults. Nature 448, 183–187 (2007). 17625563 10.1038/nature05962

[b47] Watts A. B. Gravity anomalies over oceanic rifts. Continental and Oceanic Rifts Geodynamics Series 8, 99–105 (1982).

[b48] Brink U.S.t., Coleman D. F. & Dillon W. P. The nature of the crust under Cayman Trough from gravity. Mar. Petroleum Geol. 19, 971–987 (2002).

[b49] Lambeck K. Gravity anomalies over ocean ridges. Geophys. J. Int. 30, 37–53 (1972).

[b50] Cheng Q. Mapping singularities with stream sediment geochemical data for prediction of undiscovered mineral deposits in Gejiu, Yunnan Province, China. Ore Geol. Rev. 32, 314–324 (2007).

[b51] Cheng Q. & Agterberg F. P. Singularity analysis of ore-mineral and toxic trace elements in stream sediments. Comput. Geosci. 35, 234–244 (2009).

[b52] Cheng Q. Multiplicative cascade processes and information integration for predictive mapping. Nonlin. Proc. Geophys. 19, 57–68 (2012).

[b53] Schertzer D. & Lovejoy S. Physical modeling and analysis of rain and clouds by anisotropic scaling of multiplicative processes: J. Geophys. Res. 92, 9693–9714 (1987).

[b54] Veneziano D. & Furcolo P. Multifractality of rainfall and scaling of intensity-duration-frequency curves. Water Res. Res. 38, 42-1–42-12 (2002).

[b55] Sornette D. Critical Phenomena in Natural Sciences: Chaos, Fractals, Selforganization and Disorder (2nd Edition), Springer, New York. 528pp (2004).

[b56] Malamud B. D., Turcotte D. L. & Barton C. C. The 1993 Mississippi flood: a one hundred or a one thousand year event. Environ. Eng. Geosci. v. II, 479–486 (1996).

[b57] Malamud B. D., Turcotte D. L., Guzzetti F. & Reichenbach P. Landslide inventories and their statistical properties. Earth Surf. Process. Landforms 29, 687–711 (2004).

[b58] Turcotte D. L. Fractals and Chaos in Geology and Geophysics (2nd Edition), Cambridge Univ. Press. 398pp (1997).

[b59] Cheng Q. & Agterberg F. P. Multifractal modeling and spatial statistics. Math. Geol. 28, 1–16 (1996).

[b60] Agterberg F. P. Multifractal modeling of the sizes and grades of giant and supergiant deposits. Int. Geol. Rev. 37, 1–8 (1995).

[b61] Macek W. M. & Wawrzaszek A. Multifractal two-scale Cantor set model for slow solar wind turbulence in the outer heliosphere during solar maximum. Nonlin. Proc. Geophys. 18, 287–294 (2011).

[b62] Schertzer D., Lovejoy S., Schmitt F., Chigirinskaya F. & Marsan D. Multifractal cascade dynamics and turbulent intermittency. Fractals 5, 427–471 (1997).

[b63] de Wijs H. J. Statistics of ore distribution. Part I: frequency distribution of assay values. Geologie en Mijnbouw 13, 365–375 (1951).

[b64] Mandelbrot B. B. Multifractal Measures, Especially for the Geophysicist. Pure. Appl. Geophys. 131, 5–42 (1989).

[b65] Cheng Q. Generalized binomial multiplicative cascade processes and asymmetrical multifractal distributions. Nonlin. Proc. Geophys. 21, 477–487 (2014).

[b66] McKenzie D., Jackson J. & Priestley K. Thermal structure of oceanic and continental lithosphere. Earth. Planet. Sci. Lett. 233, 337–349 (2005).

